# Clinical prediction models for bronchopulmonary dysplasia: a systematic review and external validation study

**DOI:** 10.1186/1471-2431-13-207

**Published:** 2013-12-17

**Authors:** Wes Onland, Thomas P Debray, Matthew M Laughon, Martijn Miedema, Filip Cools, Lisa M Askie, Jeanette M Asselin, Sandra A Calvert, Sherry E Courtney, Carlo Dani, David J Durand, Neil Marlow, Janet L Peacock, J Jane Pillow, Roger F Soll, Ulrich H Thome, Patrick Truffert, Michael D Schreiber, Patrick Van Reempts, Valentina Vendettuoli, Giovanni Vento, Anton H van Kaam, Karel G Moons, Martin Offringa

**Affiliations:** 1 Department of Neonatology, Emma Children’s Hospital, Academic Medical Center, Amsterdam, the Netherlands; 2 Department of Epidemiology, Julius Center for Health Sciences and Primary Care, University Medical Centre Utrecht, Utrecht, the Netherlands; 3 Department of Pediatrics, University of North Carolina, Chapel Hill, North Carolina, USA; 4 Department of Neonatology, Universitair Ziekenhuis Brussel, Brussel, Belgium; 5 NHMRC Clinical Trials Centre, University of Sydney, Sydney, Australia; 6 Division of Neonatology, Children's Hospital and Research Center Oakland, Oakland, CA, USA; 7 Neonatal Unit—Department of Child Health, St George's Hospital, London, UK; 8 Department of Neonatology, University of Arkansas for Medical Sciences, Little Rock, AR, USA; 9 Department of Surgical and Medical Critical Care, University of Florence, Florence, Italy; 10 UCL Institute of Women’s Health, University College London, London, UK; 11 Health and Social Care Research, King’s College London, London, UK; 12 Centre for Neonatal Research and Education, Schools of Anatomy, Physiology and Human Biology and Paediatrics and Child Health, University of Western Australia, Subiaco, Australia; 13 Department of Pediatrics, University of Vermont College of Medicine, Burlington, VT, USA; 14 Division of Neonatology, University Hospital for Children and Adolescents, Women's and Children's Hospital, Leipzig, Germany; 15 Department of Neonatal Medicine, Hospital Jeanne of Flanders, University hospital of Lille, Lille, France; 16 Department of Pediatrics, University of Chicago Medical Center, Chicago, IL, USA; 17 University of Antwerp and Antwerp University Hospital, Edegem (Antwerp), Belgium; 18 NICU, Department of Clinical Sciences and Community Health, Fondazione IRCCS Cà Granda Ospedale Maggiore Policlinico, Università degli Studi di Milano, Milan, Italy; 19 Division of Neonatology–Department of Paediatrics, Policlinico “A. Gemelli”-Università Cattolica S. Cuore, Rome, Italy; 20 Child Health Evaluative Sciences, Research Institute, The Hospital for Sick Children, University of Toronto, Toronto, Canada

**Keywords:** Prediction rules, Prognostic models, Calibration, Discrimination, Preterm infants, Chronic lung disease

## Abstract

**Background:**

Bronchopulmonary dysplasia (BPD) is a common complication of preterm birth. Very different models using clinical parameters at an early postnatal age to predict BPD have been developed with little extensive quantitative validation. The objective of this study is to review and validate clinical prediction models for BPD.

**Methods:**

We searched the main electronic databases and abstracts from annual meetings. The STROBE instrument was used to assess the methodological quality. External validation of the retrieved models was performed using an individual patient dataset of 3229 patients at risk for BPD. Receiver operating characteristic curves were used to assess discrimination for each model by calculating the area under the curve (AUC). Calibration was assessed for the best discriminating models by visually comparing predicted and observed BPD probabilities.

**Results:**

We identified 26 clinical prediction models for BPD. Although the STROBE instrument judged the quality from moderate to excellent, only four models utilised external validation and none presented calibration of the predictive value. For 19 prediction models with variables matched to our dataset, the AUCs ranged from 0.50 to 0.76 for the outcome BPD. Only two of the five best discriminating models showed good calibration.

**Conclusions:**

External validation demonstrates that, except for two promising models, most existing clinical prediction models are poor to moderate predictors for BPD. To improve the predictive accuracy and identify preterm infants for future intervention studies aiming to reduce the risk of BPD, additional variables are required. Subsequently, that model should be externally validated using a proper impact analysis before its clinical implementation.

## Background

Over recent decades, advances in neonatal care have improved survival amongst very preterm infants, but high rates of morbidity remain [1,2]. Bronchopulmonary dysplasia (BPD) is one of the most important complications of preterm birth and is associated with the long lasting burdens of pulmonary and neurodevelopmental sequelae [3-5].

Many interventions to reduce the risk of BPD have been tested in randomized clinical trials (RCTs), but only a few have shown significant treatment effects [6,7]. One of the possible explanations for these disappointing results may be the poor ability to predict the risk of BPD at an early stage in life, thereby failing to identify and include in RCTs those patients who will benefit most from interventions that may reduce the risk of BPD.

Developing, validating and implementing prognostic models are important as this provides clinicians with more objective estimates of the probability of a disease course (i.e. BPD), as a supplement to other relevant clinical information [8-11]. In neonatology, several studies have developed clinical prediction models, using logistic regression or consensus, to predict which preterm born infants are most likely to develop BPD [12-14]. These studies determined risk factors in a heterogeneous population of patients by using various clinical and respiratory parameters at different postnatal ages. Quantifying the predictive ability of these models in other preterm populations that were not used in the model development, often referred to as external validation of prediction models, is rarely performed. Perhaps as a consequence, none of these models have yet been implemented in clinical care to guide patient management, or used in RCTs that test interventions aimed to reduce BPD.

The primary aim of this study was to systematically review all existing clinical prediction models for BPD in the international literature, and subsequently validate these models in a large external cohort of preterm infants to determine which model yields the best prediction of BPD in very preterm infants.

## Methods

### Search methods for study identification

In April 2012, two reviewers (WO and MM) identified eligible prediction models for BPD in preterm infants using a sensitive electronic search strategy of MEDLINE, EMBASE and CINAHL. The precise search query is presented in Appendix.

The ‘prediction model’ part of this search query was rerun using a recently published highly specific and sensitive search filter [15]. We compared the yield of the original search with the rerun using this search filter in terms of citations missed and number needed to read, defined as number of citations divided by the number of eventually included research papers describing a unique study.

Included reports and the abstracts of the Pediatric Academic Societies (PAS) and the European Society for Pediatric Research (ESPR) from 1990 onwards were hand searched for additional studies not found by the initial computerized search.

### Criteria for considering studies for this review

To be included in the review, the study had to meet the following criteria: (1) it described a clinical prediction model for BPD; (2) the purpose of the model was to predict BPD in preterm infants using clinical information from the first week of life; (3) the selected predictors used were universally accessible parameters such as patient characteristics (e.g. birth weight and gestational age), respiratory support (either ventilator or non-invasive support) or blood gases. Those studies investigating the prognostic use of pulmonary function testing, ultrasonography or radiographic testing, and measurements of tracheal markers were excluded.

### Data extraction and management

The following data from all included validation and derivation studies were extracted independently by two reviewers (WO and MM): year of publication, region of origin, number of hospitals including patients for the derivation cohort, type of data collection (e.g. retrospective or prospective), period of data collection, number of predictors, patient characteristics (i.e. birth weight, gestational age, gender, inclusion of non-ventilated patients), on which postnatal day the original model was developed or validated, and the definition of BPD [e.g. oxygen dependency 28 days postnatal age (PNA) or at 36 weeks postmenstrual age (PMA)], the number of patients used for derivation of the model (not applicable for the validation studies) and the number of patients for internal and external validation when performed in the study.

The following additional items specific to the development of prognostic models were collected: modeling methods [e.g. logistic regression, by consensus, or classification and regression tree (CART) models], handling of continuous predictors and missing values, method of predictor selection, model presentation (e.g. nomogram, score chart, or formula with regression coefficients), model validation (e.g. internal and external validation), measures of calibration and discriminative ability (e.g. c-indices), classification measures (e.g. specificity and sensitivity, and positive and negative predictive values).

The original equations or score charts were used to conduct quantitative external validation in order to assess the measures of calibration and discriminative ability of the retrieved models using the empirical data at hand. The original investigators of the eligible prediction models were contacted if the manuscript did not present the intercept and predictor-outcome associations of the regression equation.

### Risk of bias assessment

In contrast to reviews of randomised therapeutic studies and diagnostic test accuracy studies, a formal guideline for critical appraisal of studies reporting on clinical prediction models does not yet exist. However, we assessed the quality of the included prediction models, assembling criteria based on two sources. First, we assembled quality criteria as published in reviews on prognostic studies [16,17]. Second, as prediction models usually come from observational studies, we used the Strengthening the Reporting of Observational Studies in Epidemiology (STROBE) [18]. This initiative developed recommendations on what should be included in an accurate and complete report of an observational study, resulting in a checklist of 22 items that relate to the title, abstract, introduction, methods, results, and discussion sections of articles. The methodological quality of the studies that developed prediction models using an observational cohort was assessed using the STROBE statement. The presence or absence of report characteristics was independently assessed by two reviewers (WO and MO). Furthermore, as recommended, the statistical methods, missing data reporting, and use of sensitivity analyses were judged. From the information in the Results and Discussion sections of each report the inclusion and attrition of patients at each stage of the study, reporting of baseline characteristics, reporting of the study’s limitations, the generalizability, and whether the source of funding was reported, were assessed and judged. High risk of bias was considered present when no descriptions of patient selection or setting, or no description of outcomes, predictors, or effect modifiers were found in the report. Unclear risk of bias was considered present when these items were described, but in an unclear manner. Otherwise low risk of bias was concluded.

### Quantifying the predictive accuracy of the retrieved models in a large independent dataset

The Prevention of Ventilator Induced Lung Injury Collaborative Group (PreVILIG collaboration) was formed in 2006 with the primary investigators of all RCTs comparing elective high frequency ventilation (HFV) with conventional ventilation in preterm infants with respiratory failure in order to investigate the effect of these ventilation strategies using individual patient data [19]. Access to and management of the individual patient data from the PreVILIG database has been described in the published protocol [20]. PreVILIG collaborators provided de-identified individual patient data to the PreVILIG Data Management Team. Access to the PreVILIG dataset was restricted to members of the PreVILIG Steering Group and Data Management Team. The original investigators continued to have control over how their data were analyzed. Newly planned analyses, such as reported in this paper, were only done if collaborators were fully informed and agreed with them.

The need for review by an ethical board has been waived. However, collaborators providing individual patient data, signed a declaration that under no circumstance patient information could possibly be linked to the patient identity.

From the 17 eligible RCTs on this topic in the literature, 10 trials provided pre-specified raw data from each individual study participant, including patients’ characteristics, ventilation parameters, early blood gas values and neonatal outcomes. These data from 3229 patients, born between 1986 and 2004, were stored in a central database. The mean gestational age of these infants was 27.3 weeks (standard deviation (SD) ±3.8 weeks) and mean birth weight was 989 grams (SD ±315 grams). External validation of the retrieved models was performed using the PreVILIG database after agreement by all the PreVILIG collaborators.

In this dataset, patient characteristics such as gestational age, birth weight, gender, Apgar score at 5 minutes and antenatal steroids were available for all infants. The median age at randomization varied between 0.3 and 13.5 hours after birth. Information on mean airway pressure (*P*_*aw*_) and the fractional inspired oxygen concentration (FiO_2_) were provided for the first 24 hours and data on ventilator settings during the first 72 hours after randomization. Data on the arterial partial oxygen tension (PaO_2_) were collected on randomization, whereas partial carbon dioxide tension (PaCO_2_) values (arterial or capillary) were available for the first 72 hours after randomization. Clinical data on surfactant use, postnatal age at randomization, and age at extubation; morbidities such as persistent ductus arteriosus, pneumothorax, pulmonary interstitial emphysema and intracranial hemorrhage; and death at 36 weeks PMA as well as the incidence of BPD defined as oxygen dependency at 36 weeks PMA were also collected. In general, the percentage of missing information from the individual patient data was low, less than 10%.

Most prediction models used conventional respiratory support in their developmental cohorts and therefore included solely conventional respiratory settings as predictor variables. The external PreVILIG cohort included infants on HFV and on conventional ventilation [19]. No apparent difference was seen in the outcome estimate BPD or the combined outcome death or BPD in the individual patient data (IPD) analysis by Cools et al. [19]. Therefore, the IPD of both intervention arms (HFV and conventional ventilation) were included in the analyses in the calculation of the prediction model. For models including predictors of conventional ventilation, only the patients in the IPD assigned to the conventional arm could be used. We assessed the discriminative performance of the included models using data of infants who were randomized to the conventional ventilation arm in a separate analysis and compared the results with the analysis of data from all infants.

### Statistical analyses

The included prediction models were validated using the reported information (i.e. regression coefficients, score charts or nomograms) by matching the predictors in each model to the variables in the PreVILIG dataset. A direct match was available in the PreVILIG dataset for most variables. When a predictor was not available in PreVILIG, we sought to replace the variable with a proxy variable. When no proxy variable was possible, we randomly substituted (e.g. imputed) the mean value reported in the literature for these predictors [21]. To prevent over-imputation this procedure was only performed when the missing predictor from the model had a low weight in the equation compared to the other predictors. If none of these methods could be applied, the clinical prediction model had to be excluded and was not tested in the external cohort.

Using these methods, we calculated the probability of developing BPD at 36 weeks PMA and the combined outcome death and BPD at 36 weeks PMA for each individual patient in the PreVILIG dataset. Although not all retrieved models were developed to predict both outcomes, the performance of all models was evaluated for both outcomes in terms of their discrimination and calibration.

First, the discriminative performance of the prediction models was quantified by constructing receiver operating characteristic (ROC) curves and calculating the corresponding area under the curves (AUC) with a 95% confidence interval. The ROC curve is commonly used for quantifying the diagnostic value of a test to discriminate between patients with and without the outcome over the entire range of possible cutoffs. The area under the ROC curve can be interpreted as the probability that a patient with the outcome has a higher probability of the outcome than a randomly chosen patient without the outcome [17].

Second, the calibration of all models was assessed. This describes the extent of agreement between the predicted probability of BPD (or the combined outcome death or BPD) and the observed frequency of these outcomes in defined predicted risk strata. Model calibration was visually assessed by constructing calibration plots and evaluating agreement between predicted and observed probabilities over the whole range of predictions [17]. As the calibration of a predictive model in an independent data set (external validation set) is commonly influenced by the frequency of the outcome in the validation set, we adjusted the intercept of each model using an offset variable in the validation data to account for prevalence differences between the populations before applying it to the data, such that the mean predicted probability was equal to the observed outcome frequency [22]. Calibration plots were constructed for the top 5 discriminating prediction models [23].

In order to determine the impact of the missing values within the PreVILIG database on the performance and accuracy of the prediction models, missing data were imputed by means of multiple imputation using “Multivariate Imputation by Chained Equations” (MICE) [24]. This procedure is an established method for handling missing values in order to reduce bias and increase statistical power [21]. Missing values were imputed 10 times for each separate trial, or, when variables were completely missing within a trial the median observed value over all trials was used. Estimates from the resulting 10 validation datasets were combined with Rubin's rule (for calculating AUCs) and with averaging of model predictions (for constructing calibration plots) [25]. Sensitivity analyses were performed to compare accuracy and calibration in validations with and without these imputed values.

All AUCs and calibration plots were constructed using R statistics (R Development Core Team (2011). R: A language and environment for statistical computing. R Foundation for Statistical Computing, Vienna, Austria). All statistical tests were conducted two-sided and considered statistically significant when *p* < 0.05.

## Results

### Literature search

**Figure 1:**
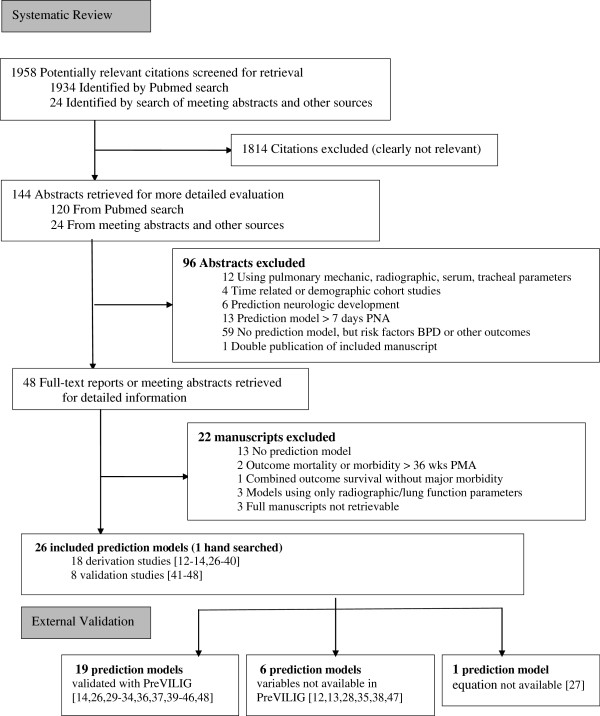
Flowchart of the systematic review of prediction models for BPD in preterm infants (updated on 01-04-2012) and the possibility of external validation using the PreVILIG dataset.

The search strategy identified 48 relevant reports (46 found on MEDLINE and 2 by hand search of the Annual Scientific Meetings, see Figure 1). Electronic searches of EMBASE, CINAHL and the CENTRAL in the Cochrane Library revealed no new relevant studies. The abstracts of these studies were reviewed independently by two reviewers (WO and MM) for inclusion in this project. After reading the full papers, 22 reports were excluded from this review for the reasons shown in Figure 1. Thirteen of the 22 excluded articles did not present a genuine prediction model, but were observational studies on risk factors for the outcome BPD.

Compared to the search query developed for the identification of prediction models in non-pediatric medicine [15], the present search strategy yielded a higher combination sensitivity and specificity by identifying 5 eligible prediction models without missing a citation, but at the expense of a higher number needed to read (NNR 93.2 vs. 74.4).

Finally, 26 study reports with publication dates ranging from 1983 to 2011 could be included in this review. Eighteen studies developed a multivariable prediction model [12-14,26-40], whereas four reported the performance of univariable parameters as a prediction model [41-44]. The remaining 4 reports [45-48] were studies validating existing prediction models originally designed for other outcomes, such as mortality [49-51]. Although developed for another outcome, these validation studies aimed to determine to which extent the prediction rule could predict BPD. Of the included reports, four studies developed a model using radiographic scoring, but also a prediction rule without this diagnostic tool and were therefore included [13,26,29,44]. Four study reports presented a prediction rule based on clinical information collected after the 7^th^ postnatal day which was beyond the scope of this review, but presented a prediction rule based on early postnatal information as well, which was included [14,30,34,40].

### Characteristics of prediction models

The models’ characteristics (Table 1) are presented for derivation studies (i.e. studies developing a novel prediction model) and validation studies (i.e. studies evaluating a single predictor or a known model for outcomes other than BPD). All models show much heterogeneity with respect to the years the data were collected, study design, total numbers of patients and gestational age. Nine of the derivation cohorts included non-ventilated patients in their developmental cohort (50%). Most studies were based on collection of data in a single-center setting. The earlier prediction models calculated their models on the outcome BPD at 28 days of postnatal age, whereas after the millennium all studies aimed for the diagnosis of BPD at the 36^th^ week PMA. Two models defined BPD according to recently established international criteria [52,53]. These models used the physiological definition at 36 weeks PMA and divided BPD into grades of severity [39,40].

**Table 1:** Characteristics of prediction models

**Study**	**Year of publication**	**Region (No. Of Centers)**	**Period of data collection**	**Study design†**	**Non-ventilated patients included**	**No. of patients derivation cohort**	**ROC timing**	**Gestational age (wks, mean ± SD)**	**Original outcome**	**Internal/ External validation**	**No. of patients validation cohort‡**
**Derivation cohorts**											
Cohen [12]	1983	USA (1)	1987-1981	Pros	No	-	2d	Un§	Death/BPD 30d	Yes/No	69/-
Hakulinen [13]	1988	Finland (1)	1978-1982	Pros	Yes	91	1d	Un§	Death/BPD 28d	No/No	−/−
Sinkin [14]	1990	USA (3)	1983-1985	Retro	Yes	160	12 h	30.9 (±4.2)	BPD 28d	Yes/Yes	49/189
Palta [26]	1990	USA (5)	-	Retro	Yes	-	3d	Un§	BPD 30d	Yes/No	42/-
Parker [27]	1992	USA (1)	1976-1990	Retro	Yes	2375	at adm.	Un§	Death/BPD 28d	Yes/No	Un§/-
Corcoran [28]	1993	UK (1)	1980-1990	Retro	No	312	3d	Un§	BPD 28d	Yes/No	100/-
Ryan 1994 [29]	1994	UK (2)	1988-1989	Retro	No	166	7d	28 (23–31)£	BPD 28d	No/Yes	-/133
Rozycki [30]	1996	USA (1)	1987-1991	Retro	No	698	8 h	Un§	BPD 28d	No/No	−/−
Ryan 1996 [31]	1996	UK (1)	1991-1992	Retro	No	202	4d	28 (23–27)£	BPD 36w	Yes/No	47/-
Romagnoli [32]	1998	Italy (1)	1989-1991	Retro	No	50	3d, 5d	28.4 (±2.2)	BPD 28d	Yes/No	149/-
Yoder [33]	1999	USA (3)	1990-1992	Pros	Yes	107	12 h, 72 h	Un§	Death/BPD 36w	Yes/Yes	54/56
Kim [34]	2005	Korea (1)	1997-1999	Retro	Yes	197	4d, 7d	28.2 (±1.9)	BPD 36w	Yes/No	107/-
Cunha [35]	2005	Brasil (1)	2000-2002	Pros	No	86	7d	27.2 (±3.2)	BPD 36w	No/No	−/−
Choi [36]	2006	Korea (Un§)	-	-	No	81	1d, 4d, 7d	Un§	BPD 36w	No/No	−/−
Henderson-Smart [37]	2006	Aus/NZ (25)	1998-1999	Pros	Yes	5599	at birth	29 (27–30)£	BPD 36w	Yes/No	5854/-
Bhering [38]	2007	Brasil (1)	1998-2003	Retro	Yes	247	7d	29.1 (±2.4)	BPD 36w	Yes/No	61/-
Ambalavanan [39]	2008	USA (16)	2001-2003	Pros	No	420	variable	26 (±2)	Death/BPD 36w	No/No	−/−
Laughon [40]	2011	USA (17)	2000-2004	Pros	Yes	2415	1d, 3d, 7d	26.7 (±1.9)	Death/BPD 36w	Yes/Yes	1214/1777
**Validation cohorts**											
Subhedar [41]	2000	UK (1)	-	Retro	No	NA	<24 h	29 (26–30)£	Death/BPD 36w	-	NA/155
Srisuparp [42]	2003	USA (1)	1996-1997	Retro	No	NA	<6 h	27.6 (±2.4)	BPD 36w	-	NA/138
Choukroun [43]	2003	France (1)	-	Retro	No	NA	at SF/18 h after	29.5 (±1.5)	BPD 36w	-	NA/44
Greenough [44]	2004	UK (1)	1998-2001	Retro	No	NA	7d	26 (24–28.6)£	Death/BPD 36w	-	NA/59
Fowlie [45]	1998	UK (6)	1988-1990	Retro	Yes	NA	72 h	29 (23–38)£	Death/BPD 36w	-	NA/398
Hentschel [46]	1998	Germany (1)	1991-1993	Retro	Yes	NA	at adm.	28.6 (±0.3)	Death/BPD 36w	-	NA/188
Chien [47]	2002	Canada (17)	1996-1997	Pros	Yes	NA	at adm.	29 (±2)	BPD 36w	-	NA/4226
May [48]	2007	UK (1)	2004-2005	Retro	Yes	NA	2d	Un§	BPD 28d/36w	-	NA/75

† Pros: prospective; retro: retrospective ‡ Number of patients in validation cohort: internal/external. §Un Unknown. ¶ Manuscripts validating the outcome BPD on models originally derived for different outcomes (e.g. mortality). £ Median gestational age (range). NA Not applicable.

### Overview of considered and selected predictors

Candidate predictors differed substantially across the identified derivation studies (Table 2), and after variable selection a median of 5 included predictors was found (range 2–12). A large proportion of the models used the infants’ gestational age and/or birth weight to calculate the risk for BPD (18 and 16 models, respectively). Gender and low Apgar scores were included in only 5 and 8 models, respectively. All multivariable models and one bivariable model used some form of the ventilator settings variable as a predictor, except for the one developed by Henderson-Smart, which only used birth weight, gestational age and gender in the equation [37]. Most models selected either the amount of oxygen administered, or the positive inspiratory pressure or mean airway pressure. A minority of the models used blood gasses at an early age as a predictor for BPD.

**Table 2:** Overview of selected and used predictors in models

**Study**	**Cohen **[12]	**Hakulinen **[13]	**Sinkin **[14]	**Palta **[26]	**Parker **[27]	**Corcoran **[28]	**Ryan 1994 **[29]	**Rozycki **[30]	**Ryan 1996 **[31]	**Romagnoli **[32]	**Yoder **[33]	**Kim **[34]	**Cuhna **[35]	**Choi **[36]	**Henderson-Smart **[37]	**Bhering **[38]	**Amblavanan **[39]	**Laughon **[40]	**Subhedar **[41]	**Srisuparp **[42]	**Choukroun **[43]	**Greenough **[44]	**Fowlie **[45]	**Hentschel **[46]	**Chein **[47]	**May **[48]	**Total % (n = 26)**
**Total number of predictors considered**	NA	19	79	NA	14	16	9	16	10	Un	7	27	20	Un	21	66	26	15	NA	19	NA	NA	41	5	12	NA	
**Total number of predictors selected**	2	5	4	8	7	8	4	5	3	3	6	8	4	3	3	4	6	6	NA	2	NA	NA	6	5	12	NA	
**Selected predictors**																											
***Clinical***																											
Gestational age			x		x	x	x	x		x	x	x	x	x	x	x		x	x		x	x	x		x		**69 (18)**
Birth weight			x	x	x	x			X	x	x	x			x		x	x	x		x	x	x	x			**62 (16)**
Small for gestational age																									x		**4 (1)**
Race/ethnicity					x													x									**8 (2)**
>15 % Birth weight loss																x											**4 (1)**
Gender					x	x									x		x	x							x		**23 (6)**
Antenatal steroids																						x					**4 (1)**
Outborn					x												x								x		**12 (3)**
Apgar score		x	x	x	x			x				x												x	x		**31 (8)**
Surfactant use								x									x					x					**12 (3)**
Patent ductus arteriosus							x			x			x			x											**15 (4)**
Fluid intake day 7													x														**4 (1)**
Lowest blood pressure																									x		**4 (1)**
Lowest temperature																									x		**4 (1)**
Urine output																									x		**4 (1)**
Respiratory distress syndrome (RDS)					x	x				x																	**12 (3)**
Severity of RDS																								x			**4 (1)**
Pneumonia		x		x		x																					**12 (3)**
Pulmonary hemorrhage				x																							**4 (1)**
Pulmonary interstitial emphysema		x		x						x																	**12 (3)**
Sepsis										x																	**4 (1)**
Seizures		x																							x		**8 (2)**
Intraventricular hemorrhage > grade II						x				x																	**8 (2)**
Congenital malformation																							x				**4 (1)**
Postnatal age at mechanical ventilation																	x										**4 (1)**
***Ventilator settings***																											
Modality												x						x							x	x	**15 (4)**
Mean FiO2										x	x	x					x	x								x	**23 (6)**
Minimum FiO2																							x				**4 (1)**
Maximum FiO2				x			x						x	x					x				x				**23 (6)**
Duration FiO2 > 0.6	x					x																					**8 (2)**
FiO2 1.0 for > 24 hr		x																									**4 (1)**
Positive inspiratory pressure (PIP)			x	x					X	x	x	x															**23 (6)**
Duration PIP > 25cmH2O						x																					**4 (1)**
Rate								x			x																**8 (2)**
Intermittent mandatory ventilation (IMV)							x		X	x														x			**15 (4)**
IMV > 24 hrs or > 2d	x															x						x					**8 (2)**
Mean airway pressure											x	x		x													**12 (3)**
Ventilator index								x																			**4 (1)**
***Laboratory***																											
pH																									x		**4 (1)**
pO2				x																					x		**8 (2)**
Oxygenation index												x					x		x	x		x					**19 (5)**
A-a DO2																			x	x							**8 (2)**
Pa/AO2																			x		x						**8 (2)**
Base excess																							x	x			**8 (2)**

NA Not applicable; Un Unknown.

### Quality and methodological characteristics model derivation

The methodological quality of derivation studies was generally poor (Table 3). Most studies used logistic regression analysis during model development. However, two studies did not employ a statistical approach and solely relied on expert opinion and consensus [12,26]. Apparent model quality was mainly degraded by categorization of continuous predictors (about 58% of the prediction models), employing unclear or naïve approaches to deal with missing values (84% of the studies did not address this issue at all), and using obsolete variable selection techniques (5 models used univariable P-values). Derived prediction models were mainly presented as an equation (11 studies). Score charts (5 studies) and nomograms (2 studies) were less common.

**Table 3:** Methodological characteristics of derivation studies

**Model development**	**Cohen **[12]	**Hakulinen **[13]	**Sinkin **[14]	**Palta **[26]	**Parker **[27]	**Corcoran **[28]	**Ryan 1994 **[29]	**Rozycki **[30]	**Ryan 1996 **[31]	**Romagnoli **[32]	**Yoder **[33]	**Kim **[34]	**Cuhna **[35]	**Choi **[36]	**Henderson-Smart **[37]	**Bhering **[38]	**Ambalavanan **[39]	**Laughon **[40]	Total % (n = 19) *
**Type of model**																			
Regression analysis		x	x		x	x	x	x	x	x	X	x	x	x	x	x	x	x	**84 (16)**
Tree/recursive partitioning																	x		**5 (1)**
Other	x			x															**11 (2)**
**Preliminary data analysis**																			
***Handling of continuous predictors***																			
Kept linear			x		x		x		x	x				x			x^†^		**41 (7)**
Categorized				x		x					X	x			x			x	**35 (6)**
Dichotomized								x		x^#^			x			x	x		**29 (5)**
***Missing values***																			
Complete case study	x					x						x			x				**21 (4)**
Imputations			x		x														**11 (2)**
Not specified		x		x			x	x	x	x	X		x	x		x	x	x	**63 (12)**
**Selection**																			
Stepwise selection			x		x			x	x		X	x	x			x	x	x	**53 (10)**
Univariate P-values		x				x	x							x	x				**26 (5)**
No selection	x			x						x									**16 (3)**
**Presentation**																			
Score chart						x				x	x	x	x			x			**26 (5)**
Nomogram								x									x		**11 (2)**
Model formula		x	x	x	x	x	x		x	x				x	x			x	**58 (11)**
**Model validation**																			
***Internal***																			
Cross-validation	x		x	x			x		x	x	x	x			x	x			**53 (10)**
Bootstrapping					x														**5 (1)**
Split sample						x												x	**11 (2)**
***External***																			
New data set			x						x		x							x	**21 (4)**
***Calibration measures***																			
Calibration Goodness of fit															x	x			**11 (2)**
Calibration plot																			**0 (0)**
Calibration intercept and slope																			**0 (0)**
***Discrimination measures***																			
ROC/area under ROC							x		x	x	x	x			x	x		x	**42 (8)**
***Classification***																			
Sensitivity/specificity	x					x	x	x	x	x	x	x		x			x		**53 (10)**
Accuracy rate		x	x																**11 (2)**
other				x	x													x	**16 (3)**

* Two models [12,13] did not include continuous estimators in their models, so handling of continuous predictor are calculated over 17 models.

# Romagnoli [32] presented 3 models, of which one dichotomized birth weight and one score chart.

† Ambalavanan [39] presented a CART model dichotomizing continuous predictor estimates in his manuscript, but provided us a model formula keeping the continuous outcomes linear.

Ten of the 19 models were only internally validated using cross-validation. This was usually achieved with a low number of included patients, except for two multicenter studies [37,40]. External validation was performed in 4 studies [14,29,33,40]. The discriminative performance of the different models was evaluated by calculating the AUC, or evaluating ROC curves or sensitivity and specificity. The reporting of calibration performance in all multivariable, bivariable and univariable prediction models was completely neglected.

**Figure 2:**
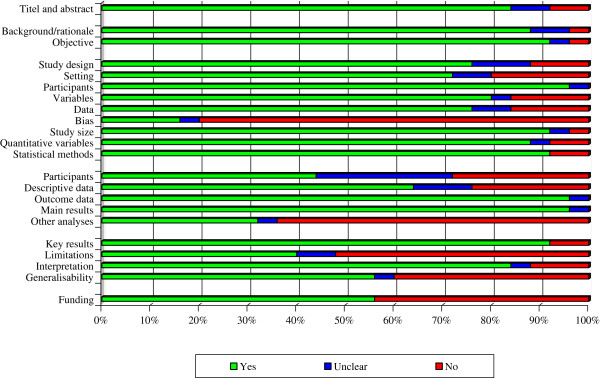
**Methodological quality of the observational cohorts according to the STROBE instrument.** Per item in the STROBE instrument, the red colour represents high risk of bias (“No”), the blue colour represents unclear risk of bias (“Unclear”), and the green colour represents low risk of bias (“Yes”).

The reporting quality of the observational studies is shown in Figure 2. There was a high correlation between the two independent assessors with only 2.7% initial disagreement (17 of of 624 scored items). These disagreements were resolved after discussion and consensus was reached.

The overall quality of the included studies was judged “high risk of bias”, “unclear risk of bias” or “low risk of bias” for all 22 items of the STROBE instrument. The individual items that were judged as high risk of bias included: lack of reporting possible sources of bias in the Methods section; not reporting actual numbers of patients in the different stages of the study; failing to report analyses of subgroups; not addressing interactions or doing sensitivity analyses. Few studies addressed their limitations and the generalizability of their results. Furthermore, nearly 50% of the studies did not report their funding source.

### External validation of the eligible models

We were able to perform external validation with the PreVILIG dataset in 19 of the 26 eligible prediction models. One study did not present the actual formula of the derived prediction model. The original investigators were not able to provide these data, and therefore its validation was not possible [27]. Two authors provided estimated predictor-outcome associations that were not described in the original reports [39,40]. One author agreed to re-analyze their data in order to construct separate models for predicting the combined outcome of death and BPD [40].

Six models could not be validated because variables on either fluid intake, weight loss after the first week of life, or exact duration of high oxygen and positive inspiratory pressure were not available in the PreVILIG dataset and no proxy variable could be imputed [12,13,28,35,38,47].

One study presented three models: a score chart, a dichotomized predictor and a model keeping all continuous variables linear [54]; the latter of these models was validated with the PreVILIG dataset [32]

The method of replacing a missing variable by a proxy was used in 3 prediction models [40,45,46]. The “base excess” values were imputed according to the mean values found in the literature [55,56]. Because subject ethnicities were not recorded in the PreVILIG validation dataset, imputation was applied on a per-trial level according to reported percentages of ethnicity. If this information was not available, the local percentage was imputed. For one model, the variable “pulmonary hemorrhage” was removed from the equation, since in the literature a negligible frequency of this complication was found, confirmed both by clinical experience and the low frequency in the original developmental cohort of this model itself [26].

### Discriminative performance

The discriminative performance of the models validated with the PreVILIG dataset (Table 4) in the complete case analyses (CCA) and multiple imputation analyses (MI) ranged from 0.50 to 0.76 for both outcomes. Regarding the outcome BPD, superior discrimination was achieved for multivariable models, with AUC values above 0.70 (CCA). The model derived by Ryan et al. in 1996 achieved the best discrimination [AUC 0.76; 95% confidence interval (CI) 0.73, 0.79], and their previous model reported in 1994 performed similarly [29,31]. Also the model of Kim et al. showed fair discrimination. These models calculate the prediction on the 7^th^ (Ryan 1994) and 4^th^ (Ryan 1996, Kim) day after birth, a relatively late stage [29,34]. Only two models that had an AUC above 0.70 in the CCA used predictors assessable on the first day of life [14,26].

**Table 4:** Areas under the ROC curve of the different prediction models

	**Original cohort**		**PreVILIG cohort**									
**Study**	**Derivation**	**Internal**	**BPD at 36 wks PMA**					**Combined Death or BPD**				
			**CCA**			**MI**		**CCA**			**MI**	
	**AUC**	**AUC**	**N**	**AUC**	**(95 % CI)**	**AUC**	**(95 % CI)**	**N**	**AUC**	**(95 % CI)**	**AUC**	**(95 % CI)**
**Multivariate models**												
Sinkin [14]	-	-	829	0.70	(0.67, 0.74)	0.68	(0.66, 0.70)	997	0.75	(0.72, 0.78)	0.74	(0.72, 0.76)
Palta [26]	-	-	346	0.73	(0.67, 0.78)	0.70	(0.68, 0.72)	401	0.74	(0.69, 0.79)	0.75	(0.73, 0.77)
Ryan 1994 [29]	0.91	0.94	2030	0.72	(0.70, 0.74)	0.66	(0.64, 0.68)	2393	0.69	(0.67, 0.71)	0.67	(0.65, 0.69)
Rozycki [30]	-	-	2429	0.54	(0.53, 0.56)	0.52	(0.50, 0.54)	2888	0.54	(0.53, 0.55)	0.55	(0.53, 0.57)
Ryan 1996 [31]	0.85	0.97	1010	0.76	(0.73, 0.79)	0.70	(0.68, 0.72)	1171	0.76	(0.73, 0.78)	0.73	(0.71, 0.75)
Romagnoli [32]	0.97	0.96	390	0.60	(0.55, 0.66)	0.62	(0.60, 0.65)	449	0.61	(0.56, 0.66)	0.65	(0.63, 0.67)
Yoder [33]	-	-	330	0.67	(0.61, 0.73)	0.68	(0.66, 0.70)	380	0.72	(0.67, 0,77)	0.73	(0.71, 0.75)
Kim [34]	0.76	0.90	322	0.71	(0.65, 0.77)	0.68	(0.66,0.70)	366	0.75	(0.70, 0.80)	0.73	(0.71, 0.75)
Choi [36]	-	-	913	0.60	(0.56, 0.64)	0.66	(0.64, 0.68)	1050	0.69	(0.66, 0,72)	0.71	(0.69, 0.73)
Henderson-Smart [37]	0.84	0.84	2128	0.65	(0.62, 0.67)	0.64	(0.62, 0.66)	2585	0.69	(0.67, 0.71)	0.69	(0.67, 0.71)
Ambalavanan [39]	-	-	795	0.61	(0.57, 0.65)	0.65	(0.63, 0.67)	891	0.66	(0.62, 0.70)	0.69	(0.67, 0.71)
Laughon [40]	0.81	0.81	801	0.70	(0.67, 0.74)	0.71	(0.69. 0.73)	960	0.74	(0.70, 0.77)	0.74	(0.72, 0.76)
Fowlie [45]	NA	NA	1006	0.64	(0.60, 0.67)	0.65	(0.63, 0.68)	1166	0.69	(0.66, 0.72)	0.69	(0.67, 0.71)
Hentschel [46]	NA	NA	1885	0.66	(0.64, 0.69)	0.65	(0.63, 0.67)	2256	0.71	(0.69, 0.73)	0.70	(0.68, 0.72)
**Bivariate models**		**External**										
May [48]	NA	0.76	1918	0.55	(0.52, 0.58)	0.54	(0.52, 0.56)	2262	0.60	(0.58, 0.63)	0.58	(0.56, 0.60)
**Univariate models**												
***Oxygenation index***												
Srisuparp [42]	NA	0.65	896	0.50	(0.46, 0.54)	0.54	(0.51, 0.56)	1029	0.53	(0.50, 0.57)	0.52	(0.52, 0.57)
Greenough [44]	NA	0.72										
***Gestational age***												
Subhedar [41]	NA	0.81	2428	0.65	(0.63, 0.68)	0.64	(0.61, 0.66)	2885	0.70	(0.68, 0.72)	0.67	(0.67, 0.70)
Choukroun [43]	NA	0.73										
Greenough [44]	NA	0.42										
***Birth weight***												
Subhedar [41]	NA	0.82	2429	0.69	(0.66, 0.71)	0.67	(0.65, 0.69)	2888	0.73	(0.71, 0.75)	0.69	(0.69, 0.73)
Choukroun [43]	NA	0.73										
Greenough [44]	NA	0.54										
***Maximum FiO2***	NA	0.66	2126	0.54	(0.52, 0.57)	0.55	(0.53, 0.57)	2511	0.60	(0.57, 0.62)	0.56	(0.56, 0.60)
***Antenatal steroids***	NA	0.54	2347	0.53	(0.51, 0.55)	0.52	(0.50, 0.54)	2800	0.50	(0.48, 0.52)	0.50	(0.48, 0.52)
***Surfactant***	NA	0.77	2429	0.55	(0.53, 0.57)	0.52	(0.51, 0.54)	2888	0.53	(0.52, 0.55)	0.53	(0.51, 0.54)
***Ventilation > 7 days***	NA	0.75	2190	0.64	(0.62, 0.66)	0.59	(0.58, 0.61)	2556	0.58	(0.57, 0.60)	0.58	(0.56, 0.59)

**CCA** Complete case analysis; **MI** Multiple imputation analyses; **AUC** Area under the curve of the receiver operator characteristics; **CI** Confidence interval; **NA** Not applicable.

Five models with the best discriminating performance for BPD showed an AUC of more than 0.70 for the combined outcome death or BPD at 36 weeks PMA [14,26,31,34,40], together with two models with a lower discriminating performance on the outcome BPD [33,46].

In contrast with predicting the outcome BPD, external validation of the univariable variables gestational age and birth weight showed an AUC ≥ 0.70 when calculated for the combined outcome death and BPD at 36 weeks PMA, underlining the weight of these two variables for the prediction of that outcome.

The range of number of patients with data on the required variables available in the PreVILIG dataset for the different models varied widely from 322 to 2429 patients. This may explain why validation results from CCA and MI sometimes considerably differed. However, multiple imputation generally resulted in a decreased AUC and these differences did not exceed 10% of the original score (Table 4). The model derived by Laughon et al. achieved the highest AUC for both outcomes, with CCA and MI [40].

The separate validation analysis of the models using only the conventionally ventilated infants in the PreVILIG dataset did not change the discriminative performance of the models under consideration, although their confidence intervals increased due to loss of power (data not shown).

### Calibration

**Figure 3:**
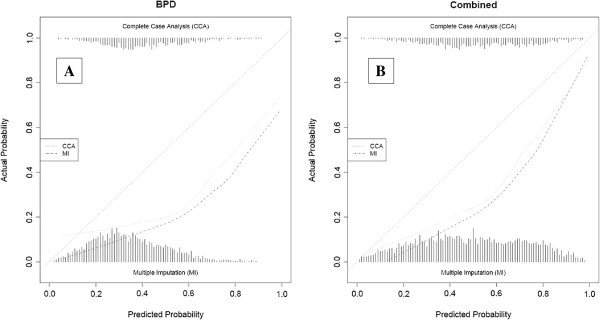
**Calibration plot of prediction model as described by Sinkin**[14]**for the outcome BPD (panel A) and the combined outcome death or BPD at 36 weeks (panel B).**

**Figure 4:**
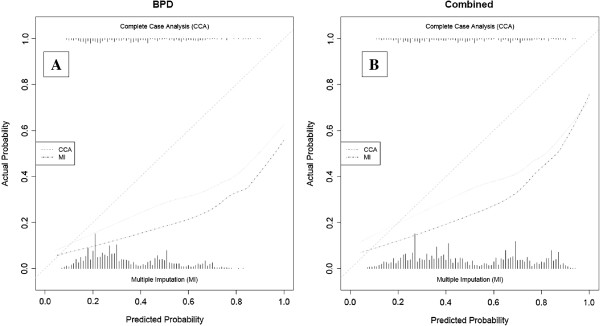
**Calibration plot of prediction models as described by Palta**[26]**for the outcome BPD (panel A) and the combined outcome death or BPD at 36 weeks (panel B).**

**Figure 5:**
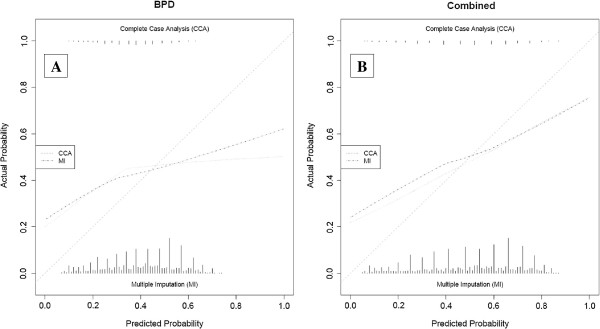
**Calibration plot of prediction model as described by Kim**[34]**for the outcome BPD (panel A) and the combined outcome death or BPD at 36 weeks (panel B).**

**Figure 6:**
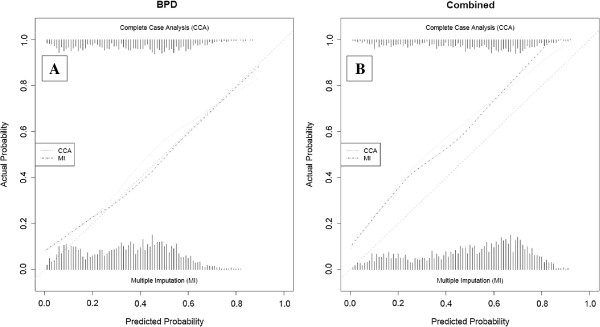
**Calibration plot of prediction model as described by Ryan 1996**[31]**for the outcome BPD (panel A) and the combined outcome death or BPD at 36 weeks (panel B).**

**Figure 7:**
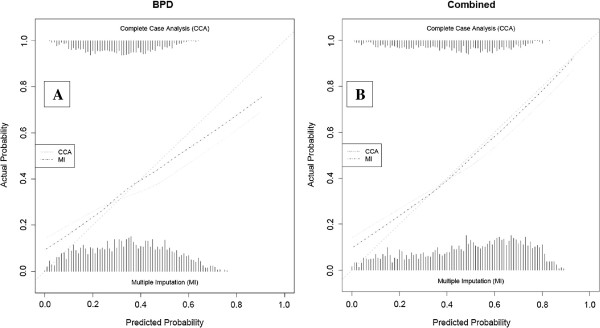
**Calibration plot of prediction models as decribed by Laughon**[40]**for the outcome BPD (panel A) and the combined outcome death or BPD at 36 weeks (panel B).**

The calibration was assessed for the 5 best-discriminating models on both the outcome BPD at 36 weeks PMA and the combined outcome death or BPD at 36 weeks PMA (CCA and MI) [14,26,31,34,40]. These plots are presented after adjustment of the intercept of each model in the validation data (Figures 3, 4, 5, 6 and 7) respectively displaying the outcome BPD (A) and the combined outcome death or BPD at 36 weeks (B)). The dashed line represents the ideal calibration (with intercept 0 and regression coefficient 1). The dotted line represents the calibration performed with complete case analysis (CCA), whereas the dash-dot line represents the multiple imputation analyses (MI). Because the incidence of BPD in the PreVILIG dataset differed from the original derivation cohorts, the calibration plots are presented with an adjusted intercept. The calibration line does not correspond well with the reference line (i.e. the predicted outcomes do not agree with the observed frequencies in all risk strata) in three of the five plots, showing both over- and underestimation by the models over the entire range of predicted probabilities (Figures 3, 4, 5, 6 and 7) [17]. The models showing good calibration are the models derived by Ryan and Laughon (Figures 6 and 7) [31,40].

## Discussion

We present the first systematic review identifying, appraising and externally validating all previously published prediction models on BPD in premature infants. We identified 26 studies published over 30 years. The external validation of 19 prediction models in the large independent PreVILIG dataset showed a broad range of discrimination performance from poor to fair. Furthermore, even with fair discriminative performance, only two models showed good calibration [31,40]. The implementation of these models in daily clinical and research practice was previously jeopardized by several limitations.

First, identified prediction models were rarely externally validated, but were predominantly evaluated by some type of internal validation. The few external validation studies done were performed in small datasets, rendering published results highly susceptible to sampling variability. Furthermore, almost all studies excluded the cases with missing values or did not specify how these cases were handled during validation [21]. As a consequence, most identified prediction models had an unclear generalizability [10]. Our multiple imputation analyses showed that only three models reached an AUC value of 0.70 for the outcome BPD, a performance statistic that is considered ‘fair’ in the literature [26,31,40], and seven models on the combined outcome death or BPD at 36 weeks PMA reached this value [14,26,31,33,34,36,40,46]. It is now widely accepted that if the value of the discriminant performance expressed in AUC ROC is less than 0.80, the model should be used with caution [57].

Second, no prediction model previously evaluated or reported its calibration. Our study demonstrates that even for models with a fair discriminative performance, calibration was far from ideal. Particularly, three of the five best discriminant models showed both over- and underestimation over the entire range of BPD probabilities [14,26,34]. One model with good calibration has a limited usefulness because it only estimates the BPD risk on the 4^th^ day of life and does not account for other forms of mechanical ventilation [31]. Another model with good calibration could not be fully evaluated because one of its predictors, HFV vs. conventional ventilation, was a randomized variable in the PreVILIG dataset [40].

Third and most importantly, none of the identified prediction models had undergone a proper implementation phase in clinical practice and research. An impact analysis is needed to quantify whether the use of the prognostic model does improve decisions and treatment and, more importantly, does improve patient outcome [11].

In summary, we conclude that although the prediction models have a reasonable quality of reporting, there are many gaps in the development, validation and impact stages of the presented models. The external validation study shows that most prediction models do not perform well enough to be considered in routine care. Out of 19 validated models, only 2 showed promising discrimination and calibration [31,40].

Several lessons can be learned from the results of this extensive validation study. In line with previous research, univariable models yield a lower discriminative ability than multivariable models. The best discriminating models selected either gestational age, birth weight, or both as a predictor. These predictors are established risk factors for BPD [52,58]. All models used respiratory/ mechanical ventilator settings as predictors, for example the concentration of supplemental oxygen or the inspiratory pressure applied to the infant. Finally, this systematic review can guide researchers in developing or updating existing prediction models following the methodology described above.

### Limitations

To fully appraise the results of our systematic review, a few important limitations need to be considered. First, of the 26 eligible models, only 19 could be validated in the PreVILIG dataset. Although it is possible that the remaining models have good performance, this remains untested so far, as some predictor variables were unavailable in the PreVILIG study. However, these variables may be difficult to assess in daily clinical practice, jeopardizing their implementation in routine care. For instance, three of the untested models included the concentration of oxygen or positive inspiratory pressure for a prescribed time [12,13,28], or included weight loss, fluid intake or urine output as predictors [35,38,47]. These variables are not collected easily even in a prospective study and more importantly are not independent of local protocols or habits [12,28].

Second, a limitation of the validation using the PreVILIG dataset is that this dataset only contains ventilated preterm infants and their available parameters during the first days of life. Today more and more infants are initially managed without invasive ventilation. Although these preterm infants often have decreased need for supplemental oxygen or mechanical ventilation in the first postnatal week, many infants have a pulmonary deterioration in the second postnatal week, with an increased need for supplemental oxygen and respiratory support, and many will eventually develop BPD [59]. Ideally, the identified prediction models should be validated using a dataset of both ventilated and non-ventilated preterm infants from a recently collected multicenter cohort, defining the outcome BPD according to recent established criteria that include the severity of the diagnosis. The PreVILIG dataset did not access the severity of BPD, and furthermore no prediction model with extensive ventilator parameters could be validated after the third day of life. However, the strength of the PreVILIG dataset is the large number of included patients, with comparable mean gestational age compared to the best five performing models, in an IPD database containing detailed information on clinical data and respiratory support during the first week after birth. Even when the limitations of this dataset are taken into account, those prediction models that have adequate generalizability should perform similarly in this dataset, as if it were a mixed dataset of both ventilated and non-ventilated infants. To assess the risk of bias due to non-randomly missing values, the calculations were rerun after multiple imputations. Overestimation of the discriminative performance due to this bias seems implausible, because these analyses showed little change in the AUC values for each model.

Third, although the appraisal of the 26 studies using the STROBE criteria showed that the quality of these studies ranged from moderate to excellent, this instrument does not estimate the quality of any prognostic study. It was developed merely to assess and improve the quality of reporting observational research [18]. Therefore, it does not include items specified for the design and conduct of prognostic research, such as selection of predictors, handling of missing values, and internal and external validation. Although a first initiative was published very recently [60], such an instrument is currently lacking. Therefore, we combined the STROBE criteria with these other aspects of prognostic studies in our evaluation.

### Implications for practice and research

The results of this systematic review have several implications for future research. First, the international research community urgently needs a quality assessment instrument aimed at prediction model studies, similar to those for the reporting of systematic reviews, randomized controlled trials, or observational studies [18,61,62]. In contrast with the former mentioned quality assessment instruments, this instrument should address not only reporting issues like the STROBE, but assess all the different aspects of the development, validation and implementation of a prediction model, as described in a series of recently published articles [8-11,60].

Furthermore, the two promising models identified in this systematic review should be confirmed by externally validation using a more recent, large multicenter cohort, preferably studied prospectively and including both ventilated and non-ventilated preterm infants at different points of postnatal life. In order to investigate potential (new) interventions for preventing BPD, prediction models should be developed at different time points after birth to facilitate the evaluation of better targeted interventions and should investigate whether risks for the outcomes BPD and the combined outcome “death or BPD” can be assessed using the same model or, instead, need separate models. These models could then be refined for example by adding genetic susceptibility as a predictor [63]. However, more research is needed to determine which of the suggested multiple candidate genes will increase accuracy of a prediction model [64]. Another improvement in the clinical prediction models could come from using birth weight Z scores in addition to gestational age instead of combining gestational age and birth weight in the model. Although these predictors are both established risk factors for BPD, combining the two might not improve the accuracy of the model due to collinearity [52,58].

Any future model should report validation analyses, showing both discriminating and calibration performance and handling missing values in the dataset by imputation, rather than exclusion [10,21,65]. If this study reveals a model with sufficient performance, an international consensus conference should be held to determine the utility of this model and, guided by this, review what variables – at any stage after birth - could improve the prediction rule without neglecting the previous model. This method is preferable to developing yet a whole new model in isolation [22]. Finally, clear impact of using that model should be provided by showing evidence that it appropriately selects candidates for preventive interventions, and future trials investigating new interventions on the important health outcome BPD.

## Conclusion

This systematic review and external validation study demonstrates that most of the numerous existing clinical prediction models for BPD cannot be used in practice because they are of low quality and their generalizability is poorly assessed. Few studies have externally validated these models, and no study previously assessed or presented model calibration. We have demonstrated that all models show poor to moderate discriminative ability and varying calibration for the prediction of the outcome BPD, with the exception of two models from Ryan and Laughon [31,40]. These deserve further evaluation and refinement. To identify very preterm infants for inclusion in future intervention studies aiming to reduce the risk of BPD, additional variables will be required to increase the predictive accuracy of these two models. Any updated model should be externally validated and put to a test of a proper impact analysis before its clinical implementation.

## Appendix

Query used for the systematic review

Electronic searches of MEDLINE (from 1966 till April 2012), EMBASE (from 1974 till April 2012) and CINAHL (from 1982 till April 2012) were performed for publications concerning prediction models for BPD in preterm infants, using the following Medical Subject Heading terms and text words:

*(“neonatal chronic lung disease” OR* “*bronchopulmonary dysplasia” OR* “*chronic lung disease of prematurity”) AND (“predict” OR” prediction” OR “predictive value” OR “prediction rule” OR “prognosis” OR “prognostic factor” OR “evaluation” OR “evaluation study” OR “risk factor” OR “risk assessment” OR “regression analysis” OR “logistic model” OR “statistical model” OR “algorithm” OR “multivariate analysis” OR” predictive value of tests” OR “Area Under Curve” OR “Receiver Operator Curve”).* No search limits were used.

## Abbreviations

AUC: Area under the curves; BPD: Bronchopulmonary dysplasia; CCA: Complete case analyses; IPD: Individual patient data analysis; HFV: High frequency ventilation; MI: Multiple imputation analyses; PMA: Postmenstrual age; PreVILIG: Prevention of ventilator induced lung injury collaborative group; RCTs: Randomized controlled trials; ROC: Receiver operating characteristic; SD: Standard deviation; STROBE: Strengthening the reporting of observational studies in epidemiology.

## Competing interests

The authors declare that they have no competing interests.

## Authors’ contributions

WO, FC, AvK, KGM, and MO designed and initiated the study. WO, ML, MM, LA, JA, SC, SC, CD, DD, NM, JP, JP, RS, UT, PT, MS, PVR, VV, and GV were responsible for the acquisition of data. WO, TD, KGM, and MO were responsible for the statistical analysis. WO drafted the initial report. TD, ML, MM, FC, LA, JA, SC, SC, CD, DD, NM, JP JP, RS, UT , PT, MS, PVR, VV, GV, AvK, KGM, and MO made a critical revision of the manuscript for important intellectual content. FC, AvK, KGM, and MO supervised the study. WO and TD have full access to all the data in the study and take responsibility for the integrity of the data and accuracy of the data analysis. All authors approved the final manuscript.

## Pre-publication history

The pre-publication history for this paper can be accessed here:

http://www.biomedcentral.com/1471-2431/13/207/prepub
